# Altered Root Growth, Auxin Metabolism and Distribution in *Arabidopsis thaliana* Exposed to Salt and Osmotic Stress

**DOI:** 10.3390/ijms22157993

**Published:** 2021-07-27

**Authors:** Ana Smolko, Nataša Bauer, Iva Pavlović, Aleš Pěnčík, Ondřej Novák, Branka Salopek-Sondi

**Affiliations:** 1 Department for Molecular Biology, Ruđer Bošković Institute, Bijenička Cesta 54, 10000 Zagreb, Croatia; ana.smolko@irb.hr (A.S.); iva.pavlovic@irb.hr (I.P.); 2 Department for Molecular Biology, Faculty of Science, University of Zagreb, Horvatovac 102, 10000 Zagreb, Croatia; nbauer@biol.pmf.hr; 3 Laboratory of Growth Regulators, Faculty of Science of Palacký University & Institute of Experimental Botany of the Czech Academy of Sciences, Šlechtitelů 27, CZ-783 71 Olomouc, Czech Republic; ales.pencik@upol.cz (A.P.); novako@ueb.cas.cz (O.N.)

**Keywords:** abiotic stress, *Arabidopsis thaliana*, auxin distribution, auxin metabolome, auxin transcriptome, root growth

## Abstract

Salt and osmotic stress are the main abiotic stress factors affecting plant root growth and architecture. We investigated the effect of salt (100 mM NaCl) and osmotic (200 mM mannitol) stress on the auxin metabolome by UHPLC-MS/MS, auxin distribution by confocal microscopy, and transcript levels of selected genes by qRT-PCR in *Arabidopsis thaliana* ecotype Columbia-0 (Col-0) and *DR5rev::GFP* (DR5) line. During long-term stress (13 days), a stability of the auxin metabolome and a tendency to increase indole-3-acetic acid (IAA) were observed, especially during salt stress. Short-term stress (3 h) caused significant changes in the auxin metabolome, especially NaCl treatment resulted in a significant reduction of IAA. The data derived from auxin profiling were consistent with gene expressions showing the most striking changes in the transcripts of *YUC*, *GH3*, and *UGT* transcripts, suggesting disruption of auxin biosynthesis, but especially in the processes of amide and ester conjugation. These data were consistent with the auxin distribution observed in the DR5 line. Moreover, NaCl treatment caused a redistribution of auxin signals from the quiescent center and the inner layers of the root cap to the epidermal and cortical cells of the root elongation zone. The distribution of PIN proteins was also disrupted by salt stress; in particular, PIN2 was suppressed, even after 5 min of treatment. Based on our results, the DR5 line was more sensitive to the applied stresses than Col-0, although both lines showed similar trends in root morphology, as well as transcriptome and metabolome parameters under stress conditions.

## 1. Introduction

As sessile organisms, plants have amazing adaptability to the ever-changing environment and are able to survive under often unpleasant conditions such as extreme temperatures, lack of water, increased soil salinity, etc. Understanding the mechanisms of adaptation to abiotic stress has been of great importance in the last decades in the context of the current climate changes and the predicted further warming and decrease in precipitation, especially in the arid and semi-arid Mediterranean region [1].

The plant root is very sensitive to the environment and is the first plant organ to be confronted with unpleasant environmental changes in the soil such as water deficit and increased salinity. Root responses to abiotic stress are very dynamic and complex and involve high plasticity in root growth and architecture. Root system architecture (RSA) is determined by primary root growth (PR), initiation and emergence rates for lateral root (LR) primordia, and growth of LR. Root growth and RSA are processes that are strongly regulated by the phytohormone auxin. The high auxin concentration in root quiescent cells and in the stem cell niche is essential for the coordination and establishment of the different root tissues and root growth [2,3]. Maintenance of the auxin maximum in the root tip is achieved by transport of shoot-derived auxin into the root tip, where complex patterns of auxin efflux carriers lead to auxin accumulation in the stem cell niche [4]. Moreover, local auxin biosynthesis is possible in all parts of the root system [5]. The amount of auxin in a particular cell type is crucial for cell fate. Thus, the accumulation of auxin in pericycle cells transforms them into lateral root founder cells, which further divide and form a lateral root primordium [6]. 

**Figure 1:**
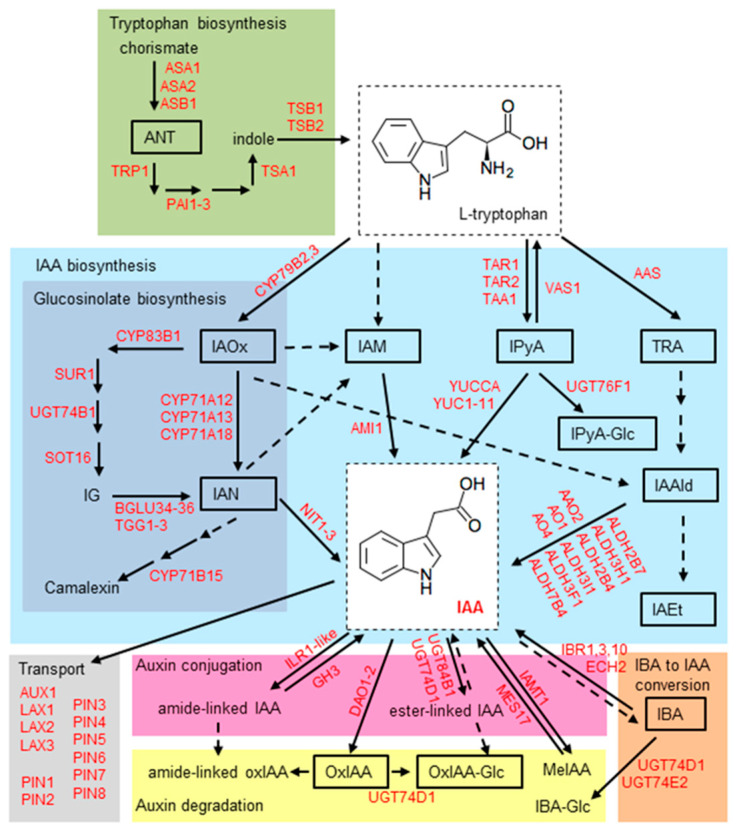
Scheme of IAA metabolism in *Arabidopsis thaliana* (Arabidopsis). The pathways involved in auxin metabolism in Arabidopsis are based on the KEGG pathways [13] and data published in [7,14,15,16,17,18,19]. The darker blue panel shows pathways specific for Arabidopsis, *Brassica,* and *Sinapis* [20]. Yellow and pink panels indicate pathways for IAA degradation [14] and IAA conjugation [15], respectively. The contribution of the IBA to IAA conversion to the active IAA pool is illustrated by the orange panel [16]. The green panel shows the synthesis of tryptophan from the precursor chorismate via the shikimate pathway [21]. Dashed arrows indicate pathways that may contain multiple enzymatic steps, and where the genes/enzymes are not known. ANT, anthranilate; IAA, indole-3-acetic acid; IAAld, indole-3-acetaldehyde; IAEt, indole-3-acetethanol; IAM, indole-3-acetamide; IAN, indole-3-acetonitrile; IAOx, indole-3- acetaldoxime; IBA, indole-3-butyric acid; IBA-Glc, indole-3-butyryl-1-*O*-β-d-glucose; IPyA, indole-3-pyruvic acid; MeIAA, indole-3-acetic acid methyl ester; oxIAA, 2-oxoindole-3-acetic acid; oxIAA-Glc, 2-oxoindole-3-acetyl-1-*O*-β-d-glucose; TRA, tryptamine.

The major processes controlling root growth and development, such as cell division, elongation, and differentiation, are mediated by the formation of local auxin maxima and local auxin minima in root tissues. Dynamic auxin gradients essential for the integration of various environmental and endogenous signals are regulated by de novo auxin biosynthesis, hydrolysis of auxin conjugates, polar transport, auxin inactivation through various conjugation and oxidation pathways. Auxin metabolism and homeostasis have been extensively studied and numerous genes have been identified to date. A review of auxin homeostasis has been updated [7,8,9,10,11,12] and is summarized in Figure 1. 

Polar auxin transport enables the adequate distribution of auxin from the site of synthesis to the site of action, thus contributing to auxin homeostasis. Components that control auxin transport are the auxin influx carriers localized in the plasma membrane, the auxin permeases of the AUX1/LAX family, and efflux facilitators and transporters, the PIN family and ABCB family, respectively [22,23].

In addition to the role of auxin in regulating root growth and development under optimal conditions, which has been well studied previously (see review [5,6]), recent data show that auxin plays an important role in mediating changes in the root during abiotic stresses (see reviews [4,24,25]). Here, we focus on salinity and osmotic stress and their effects on root growth and RSA. Salinity stress is a complex stress consisting of ionic stress caused by toxic ion concentrations (mainly Na^+^), osmotic stress caused by the associated decreased water uptake, and oxidative stress caused mainly by the increase in reactive oxygen species levels (ROS) [26]. There is a partial overlap with drought responses due to the osmotic component. However, the changes in RSA show clear differences in PR growth, which is strongly promoted during drought, whereas it is inhibited during salt stress [4].

Studies in a variety of plant species have shown that auxin homeostasis is a direct link between growth regulation and stress adaptation responses (see review [27]). According to microarray data tracking the expression of *YUCCA* flavin monooxygenases (YUCs) (involved in the major IAA biosynthetic pathway in Arabidopsis), auxin biosynthesis is shifted from columella cells to the root epidermis during salt stress [4]. This redistribution of auxin maxima correlates with reduced growth of PR and LR and a decrease in the number of LRs [28,29,30]. In *Brassica rapa* seedlings, IAA precursor profiling showed that salinity stress activates the indole-3-acetamide (IAM) and indole-3-acetaldoxime (IAOx) biosynthetic pathways, while the indole-3-pyruvic acid (IPyA) pathway is repressed [31]. It was shown that changes in CYP79B2 expression under salt stress positively correlated with the development of lateral roots in Arabidopsis, and *cyp79b2 cyp79b3* double mutants developed fewer and shorter lateral roots under salt stress compared to control conditions, also suggesting that the IAOx biosynthetic pathway is more dominant under salt stress in Brassicaceae [32]. 

In addition to auxin biosynthesis, increased salinity and osmotic stress affect polar auxin transport. It has been shown that the root bends away from the salt under a saline environment (halotropism), which is caused by redistribution of auxin supported by changes in auxin transporters (AUX1, PIN1, PIN2) [24,33]. In addition, salt exposure reduces the size of the root meristem and inhibits the formation of LR due to decreased levels of PIN1, PIN3 and PIN7 proteins [34]. Auxin transport is also inhibited during drought stress by inhibiting *PIN1* expression, which could facilitate increased downward bending of roots [34].

Looking at IAA conjugation, all GH3 enzymes involved and some UGTs seem to be highly expressed in roots and are upregulated during salt stress [4]. The oxidation process of IAA is very active in root tissues; the enzyme DAO1 catalyzes the temporal and tissue-specific oxidative inactivation of IAA, and the *DAO1* gene is expressed in the cortical, endodermal, and pericycle cells of the distal elongation zone and maturation zone of the root, root columella, and atrichoblasts, while *DAO2* is weakly expressed in the root columella and procambium of the root maturation zone [35]. The same authors showed that the *DAO2* knockdown exhibited increased lateral root density, indicating a possible role of IAA oxidation in the root response to salt stress [35]. 

Root growth is remodeled under salt and osmotic stress and the mechanisms of this remodeling are still unclear. In this work, we investigated the auxin metabolome in Arabidopsis (Col-0 and DR5 line) under short- and long-term abiotic stress conditions with NaCl and mannitol as salt and osmotic stressors, respectively. In addition, auxin distribution in root tissue of the DR5 line was examined after short-term (1.5–12 h) stressor treatments (salt and mannitol). As salt induced a more drastic redistribution of auxin, we further examined the stress-induced distribution of PIN proteins (PIN2, PIN4, and PIN7) using the Arabidopsis transgenic lines *pPIN2::PIN2-GFP*, *pPIN4::PIN4-GFP*, and *pPIN7::PIN7-GFP* at 5 to 15 min after salt treatments. Finally, the expression of auxin related genes was analyzed and root growth, auxin metabolome data, and auxin distribution patterns were discussed in correlation with the transcript level of selected genes under saline and osmotic conditions.

## 2. Results and Discussion

### 2.1. Root Growth under Abiotic Stress

**Figure 2:**
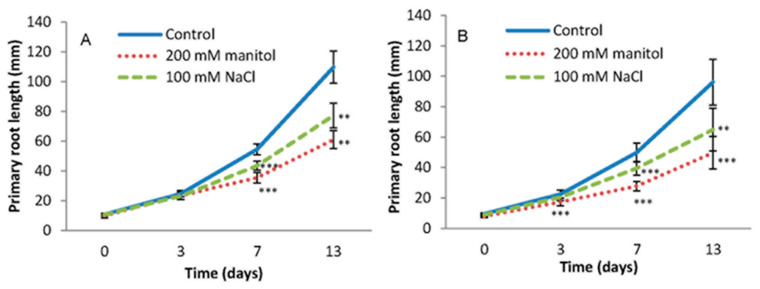
Inhibition of the primary root length of *Arabidopsis thaliana* Col-0 (**A**) and DR5 (*DR5rev::GFP*) line (**B**) grown on MS medium (Control) or MS medium supplemented with 100 mM NaCl (NaCl) and 200 mM mannitol (Man). Primary root length was monitored for 13 days. The experiment was performed on three biological replicates, each consisted of 5 roots. Results are average ± SD, *n* = 15 seedlings. Asterisks indicate significant difference in primary root length in plants grown under stress conditions versus the corresponding control in a Student’s *t*-test (** and *** correspond to *p*-values of 0.01 > *p* > 0.001 and *p* < 0.001, respectively).

To investigate the effect of salt and osmotic stress on root growth, Arabidopsis Col-0 and the DR5 lines were used. Seedlings were germinated on MS medium for 4 days and then transferred to the same medium supplemented with 100 mM NaCl or 200 mM mannitol for the next 13 days, with root growth monitored periodically. Both lines showed similar responses to the applied stressors (Figure 2, Figure S1).

There were no visible differences in root growth and morphology for the first three days of treatments in Col-0, while DR5 showed a statistically significant reduction in root length after the treatments compared to the corresponding controls. The inhibition of primary root growth was more pronounced with the duration of treatments. The primary root length was inhibited by 44.4% and 29.6% in Col-0 and 48.3% and 32.5% in the DR5 line after 13 days of treatments with mannitol and NaCl, respectively, compared to the controls (Figure 2). 

**Figure 3:**
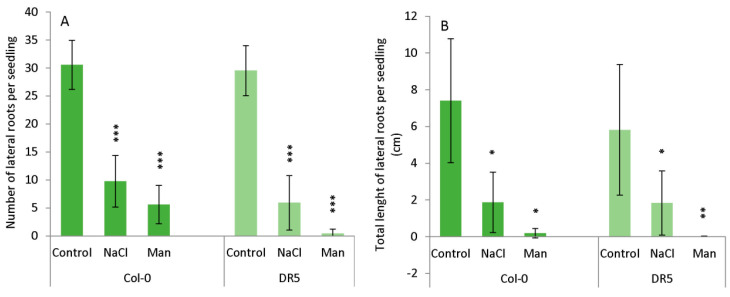
Lateral root (LR) number (**A**) and total length of LRs (**B**) in wild type *Arabidopsis thaliana* Col-0 and *DR5rev::GFP* line (DR5) upon 13-day growth on MS medium (Control) or MS medium supplemented with 100 mM NaCl (NaCl) or 200 mM mannitol (Man). The experiment was performed on five biological replicates, each consisted of 5 roots. Results are expressed as mean ± SD, *n* = 25 seedlings. Asterisks indicate significant difference in the measured parameters in controls compared to stresses conditions in each line in a Student’s *t*-test (*, **, and *** correspond to *p*-values of 0.1 > *p* > 0.01, 0.01 > *p* > 0.001, and *p* < 0.001, respectively).

The occurrence of lateral roots (LRs) was significantly reduced in NaCl- and mannitol-exposed seedlings during the studied period (Figure 3, Supplementary Figure 1). The average number of lateral roots was similar in both Arabidopsis lines under control conditions. Under salinity stress, the number of LRs in Col-0 and DR5 was significantly lower than in the corresponding controls. The effect of osmotic stress was even more drastic in both lines. The average total length of LRs also decreased under stress conditions in both lines, with osmotic stress having a greater effect (Figure 3). Thus, results indicate that stress conditions caused a similar inhibitory effect to LR numbers and total LR length in both lines.

### 2.2. Auxin Profile in Arabidopsis after Short-Term and Prolonged Stress Conditions 

**Figure 4:**
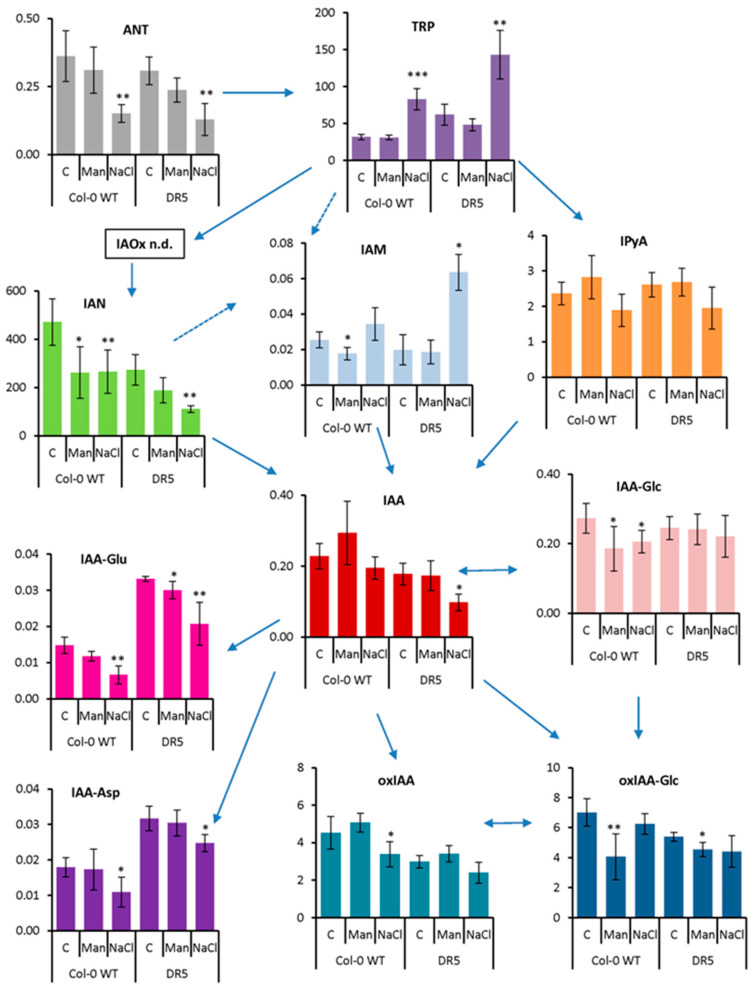
Auxin metabolome profile measured in 4-day-old seedlings of *Arabidopsis thaliana* Col-0 and *DR5rev::GFP* line (DR5) treated with 200 mM mannitol (Man) or 100 mM NaCl (NaCl) for 3 h compared to the corresponding controls (C). Metabolite levels are expressed in nmol g^−1^ FW. Results are average ± SD, *n* = 3. Asterisks indicate significant difference in the levels under stress conditions versus the control in a Student’s *t*-test (*, **, and *** correspond to *p*-values of 0.05 > *p* > 0.01, 0.01 > *p* > 0.001, and *p* < 0.001, respectively). ANT, anthranilate; IAA, indole-3-acetic acid; IAA-Asp, indole-3-acetyl-L-aspartic acid; IAA-Glc, indole-3-acetyl-1-*O*-β-d-glucose; IAA-Glu, indole-3-acetyl-L-glutamic acid; IAM, indole-3-acetamide; IAN, indole-3-acetonitrile; IAOx, indole-3-acetaldoxime; IPyA, indole-3-pyruvic acid; oxIAA, 2-oxoindole-3-acetic acid; oxIAA-Glc, 2-oxoindole-3-acetyl-1-*O*-β-d-glucose; TRP, tryptophan; n.d., not detected.

**Figure 5:**
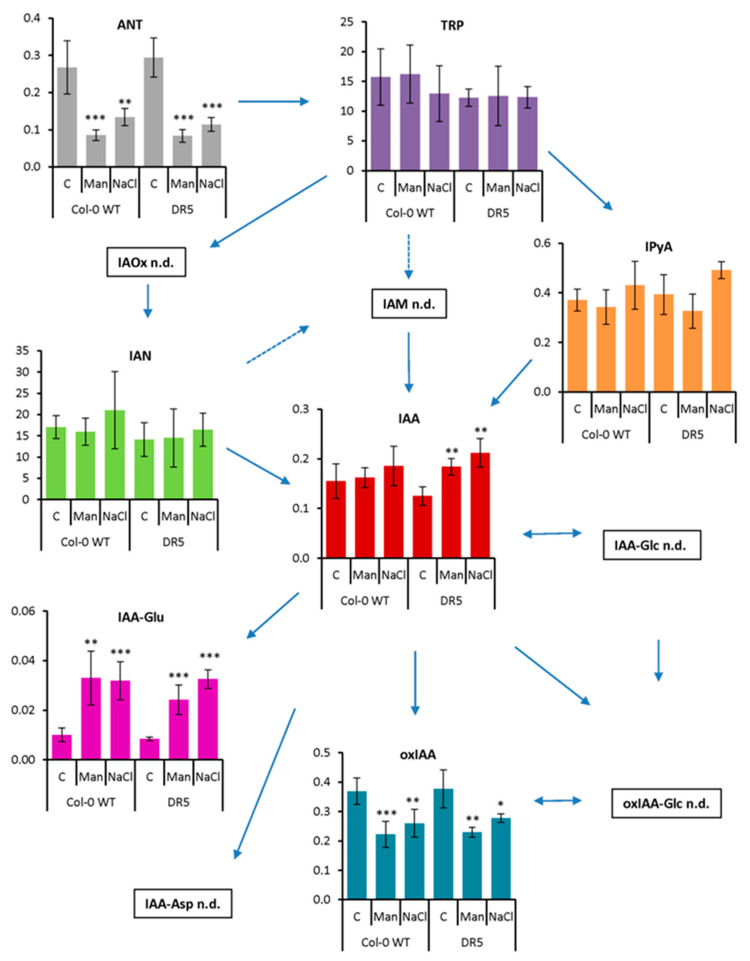
Auxin metabolome profile measured in the roots of *Arabidopsis thaliana* Col-0 and *DR5rev::GFP* line (DR5) after 13-day treatment with 200 mM mannitol (Man) and 100 mM NaCl (NaCl) compared to the corresponding control (C). Metabolite levels are expressed in nmol g^−1^ FW. Results are average ± SD, *n* = 3. Asterisks indicate significant difference in the levels under stress conditions versus the control in a Student’s *t*-test (*, **, and *** correspond to *p*-values of 0.05 > *p* > 0.01, 0.01 > *p* > 0.001, and *p* < 0.001, respectively). Abbreviations: ANT, anthranilate; IAA, indole-3-acetic acid; IAA-Asp, indole-3-acetyl-L-aspartic acid; IAA-Glc, indole-3-acetyl-1-*O*-β-d-glucose; IAA-Glu, indole-3-acetyl-L-glutamic acid; IAM, indole-3-acetamide; IAN, indole-3-acetonitrile; IAOx, indole-3-acetaldoxime; IPyA, indole-3-pyruvic acid; oxIAA, 2-oxoindole-3-acetic acid; oxIAA-Glc, 2-oxoindole-3-acetyl-1-*O*-β-d-glucose; TRP, tryptophan; n.d., not detected.

It is well known that the plant hormone auxin is crucial for all aspects of plant development, both under optimal and stress conditions [36], especially for root growth and RSA [4,5,6]. Therefore, we performed auxin metabolomic analyses by liquid chromatography-tandem mass spectrometry (LC-MS/MS) in both Col-0 WT and DR5 lines in 4-day-old seedlings after 3 h (Figure 4) and in Arabidopsis roots after 13 days of stress application (Figure 5). As can be seen in Figure 4 and Figure 5, the changes in auxin metabolites in both Arabidopsis lines followed the same trend, although the concentrations of certain metabolites differed.

After short-term stress application (Figure 4), IAA content did not change under osmotic stress but decreased under salinity stress compared with the corresponding control, especially in the DR5 line (1.8-fold). Similarly, the content of ANT, the precursor of tryptophan (TRP), was significantly decreased under salinity treatment (2.4-fold in both lines), whereas TRP was significantly increased (2.6- and 2.3-fold in Col-0 and DR5 lines, respectively). The major IAA precursor, IPyA, was not significantly affected, although there is a tendency to decrease under salinity treatment in both lines. Interestingly, several other IAA biosynthetic pathways appear to be more strongly induced under salinity stress conditions. Similar to IAA, the levels of IAN were decreased under both stress conditions, but most significantly under salinity treatment (1.7- and 2.5-fold in Col-0 and DR5 lines, respectively). However, the IAM content was increased about 3-fold under salinity stress in the DR5 line compared with the control. Auxin metabolite content was also negatively affected after short-term stress application. The levels of both amino acid conjugates, IAA-aspartate (IAA-Asp) and IAA-glutamate (IAA-Glu) were significantly decreased upon salinity stress in both lines. Interestingly, the DR5 line contained approximately 2-fold higher levels of IAA-Asp and IAA-Glu under control conditions compared with Col-0. The auxin catabolites oxIAA and its glucosyl ester (oxIAA-Glc) were each slightly decreased under salinity and osmotic stress. IAA glucosyl ester (IAA-Glc) also changed significantly in Col-0 under both stress treatments, whereas it remained unchanged in the DR5 line.

As RSA was severely affected by the prolonged osmotic and salinity stress (Figure 2 and Figure 3), we also performed auxin metabolome analysis in the roots of the 13-day-treated Arabidopsis plants and compared them with the auxin metabolome under the control conditions (Figure 5). 

Our results show that the content of ANT in both lines was significantly decreased in both prolonged stress treatments, while the content of TRP did not change. However, two detected IAA precursors, IAN and IPyA, showed a tendency to increase in NaCl treatments, although the changes were not statistically significant. Similarly, IAA also showed a tendency to increase in both lines under both stress conditions, but statistically significant changes were obtained only in the DR5 line compared with the control. These results are consistent with an increase in IAA concentration in Chinese cabbage seedlings after 24 h of salinity stress [31]. Importantly, the process of amide conjugation was strongly induced under both stress conditions. The IAA-Glu levels were significantly increased, indicating active auxin homeostasis to regulate optimal IAA levels. On the other hand, the oxIAA levels were significantly decreased under osmotic and salinity stress conditions in both lines. Furthermore, glucosyl ester conjugates were not detected after long-term stress application. Similar patterns of IAA-Glu and oxIAA concentrations were previously published in root and shoot tissues of control and salt-stressed Arabidopsis seedlings [37].

### 2.3. Early Auxin Redistribution in Root under Stress Conditions

**Figure 6:**
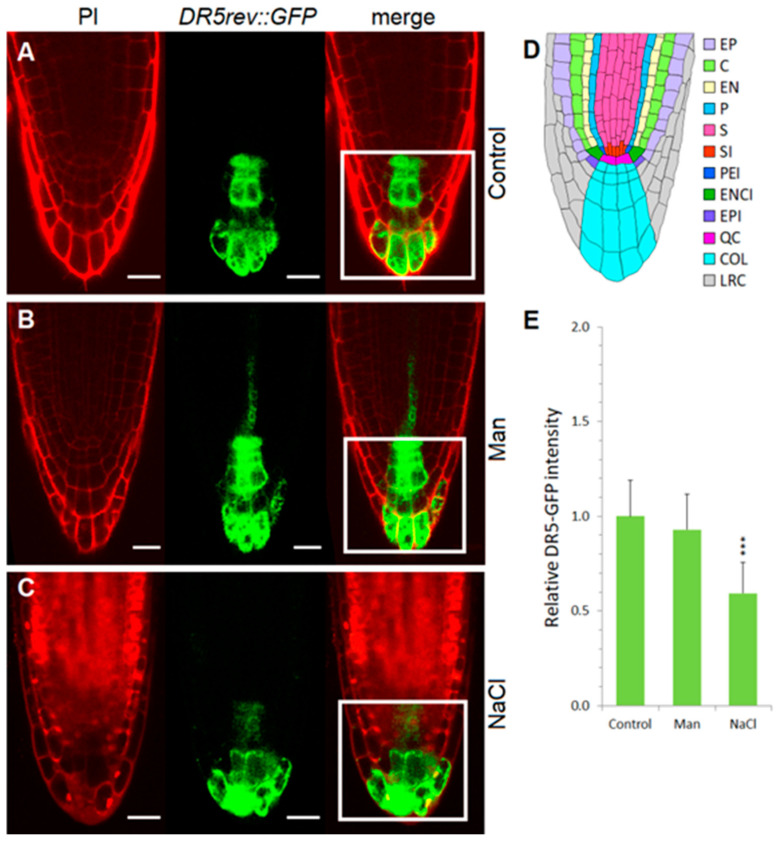
Auxin distribution and relative GFP intensity in the root tips of *DR5rev::GFP line*. Seedlings were exposed for 3 h to 2.5 mM MES (control) (**A**) or control medium was supplemented with 200 mM mannitol (Man) (**B**) or 100 mM NaCl (NaCl) (**C**). GFP fluorescence was monitored with the laser scanning confocal microscope and its intensity was quantified. The anatomy of the root tip is shown in (**D**). The area of interest was selected with the ImageJ rectangle ROI selection tool (white rectangle) in at least 10 roots. Fluorescence intensity (**E**) of these ROIs was measured as integrated density and the relative intensity, presented in the histogram, was calculated as the value of integrated density normalized to control without stressor; error bars represent SD. Asterisks (***) indicate significant differences with respect to the corresponding control (Student’s *t* test, *p* < 0.001). Abbreviations: PI, propidium iodide; E, epidermis; C, cortex; EN, endodermis; P, pericycle; S, stele; SI, stele initials; PEI, pericycle initials; ENCI, endodermis/cortex initials; EPI, epidermis initials; QC, quiescent center; COL, columella; LRC, lateral root cap. Bar: 20 µm.

Since we obtained the changes in the auxin metabolome in Arabidopsis after only 3 h of stress application, we further followed auxin distribution in the roots of 4-day-old seedlings in the range of treatments from 1.5 to 12 h by using the artificial reporter line *DR5rev::GFP* (DR5), which is the most commonly used tool for monitoring auxin distribution in planta. The most striking changes were observed in the salinity treatment, while the mannitol treatment did not cause significant auxin redistribution in the root compared with the corresponding control. This observation was in agreement with LC-MS/MS measurements under short-term stress, which also indicated less pronounced changes in the auxin metabolome under osmotic than under salinity stress (Figure 4). In control and mannitol-treated seedlings, auxin signals were concentrated in the quiescent center and in the internal layers of the root cap after 3 h (Figure 6, Video S1). 

**Figure 7:**
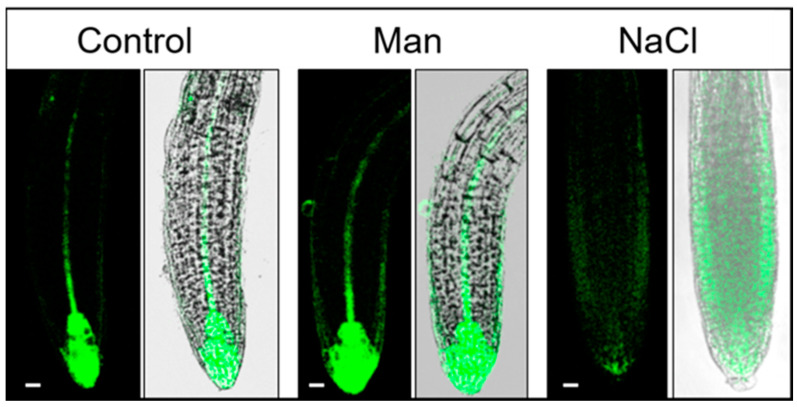
Auxin distribution in the Arabidopsis root of the DR5 line 6 h after treatment. Seedlings were exposed to 2.5 mM MES (control) or control medium supplemented with 200 mM mannitol or 100 mM NaCl. GFP fluorescence was imaged with a Leica TCS SP8 X laser scanning confocal microscope. Bar: 10 μm.

Exposure to 100 mM NaCl for 3 h caused a significant reduction of auxin in the quiescent center and in the internal layers of the root cap (Figure 6), whereas an increase of auxin was detected in the epidermal and cortex cells of the root elongation zone (Figure 7, Video S3). A similar observation was reported by Wang et al. [28] using the *DR5::uidA* line under prolonged (48 h) salt treatment (150 mM NaCl); where GUS expression is mainly distributed in the epidermal cells of the root, while the level of GUS staining is significantly reduced in the area around the quiescent center. Interestingly, as shown here, the redistribution of auxin under salinity stress is much faster than previously reported [28]. At the same time point, osmotic stress did not alter auxin accumulation or distribution compared to the corresponding control. Osmotic stress induced by mannitol increased auxin accumulation in the epidermis and provascular tissue, but auxin remained high in the quiescent center and columella (Figure 6, Video S2).

### 2.4. PINs Redistribution under Salinity Stress

As salt stress drastically affected the redistribution of auxin in different cell layers of the root elongation zone, we additionally investigated the occurrence of PINs in roots upon salt application. Previously, it was reported that reduced auxin accumulation in roots subjected to salt stress could be due to the suppression of auxin carriers in response to salt concentration and duration of stress application [34]. Most of the PIN proteins are located in the plasma membrane of root cells and are restricted to specific areas of the cell that mediate directional auxin fluxes within the tissue, influencing auxin distribution and thus root growth and architecture [38]. To investigate the effect of salt on the redistribution of PIN proteins, we used the transgenic Arabidopsis lines *pPIN2::PIN2-GFP*, *pPIN4::PIN4-GFP*, and *pPIN7::PIN7-GFP*.

**Figure 8:**
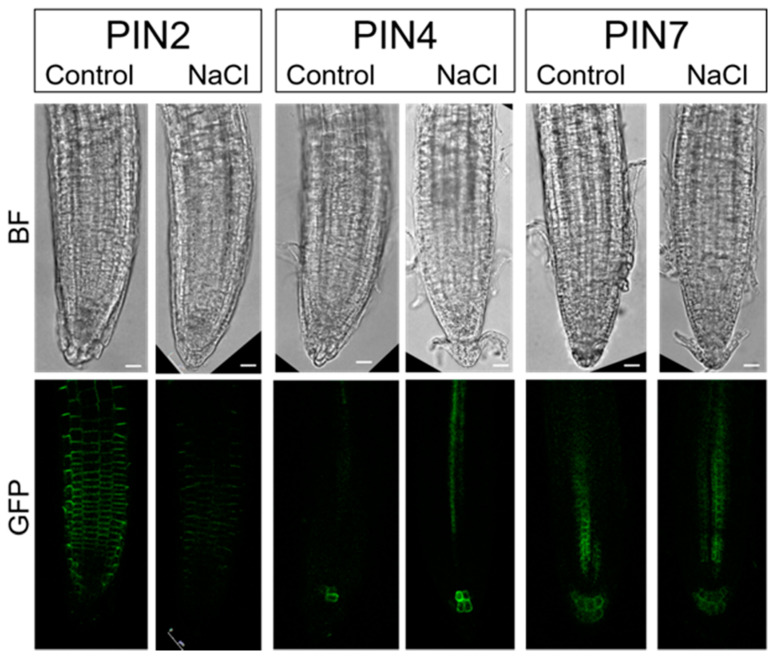
PIN proteins (PIN2, PIN4, and PIN7) distributed in the root tips of Arabidopsis *pPIN2::PIN2-GFP*, *pPIN4::PIN4-GFP*, and *pPIN7::PIN7-GFP* lines before salt stress application (Control) and in response to immediate (10 min) salt stress (100 mM NaCl). GFP fluorescence was imaged with a laser scanning confocal microscope. Bar: 10 μm.

PIN2 is known to be localized to the underside of root cortical cells and to the upper surface of epidermal cells and to act primarily in the redistribution of auxin during root gravitropism. Immediately after NaCl application, PIN2 levels decreased and remained reduced during the next 15-min of treatment (Figure 8, Figure S2). This was consistent with the results obtained on the DR5 line after NaCl treatment, which showed auxin accumulation in the root cortex and epidermis (Figure 6). These results suggest a crucial role of relocalization of PIN2 protein in the overaccumulation of auxin in the root cortex and epidermis after initial salt stress. These results are consistent with the data of Galvan-Ampudia and Testerink [33] who indicated that downregulation of PIN2 is induced when the intracellular Na^+^ level in the root increases, resulting in decreased basipetal auxin transport in the root epidermis and lateral root cap. A similar observation of a more diffuse distribution of PIN2 is reported for 12 to 24 h of salt-treated Arabidopsis roots [39].

As we found reduced auxin levels in and around the quiescent center of the DR5 line after salt stress, we also examined PIN4 and PIN7, which were previously reported to direct auxin toward the quiescent center. PIN4 is reported to localize to the basal side of cells in the central root meristem and with less pronounced polarity in the cells of the quiescent center while PIN7 is localized to the basal side of the stele cells and apolar in columella cells [40]. However, there was no change in the localization and intensity of PIN4 and PIN7 around the quiescent center immediately after salt treatment (Figures S3 and S4). Thus, the reduced auxin level in the quiescent center after short-term stress application is probably not associated with PIN4 and PIN7. However, immediately after salt stress application, we detected a stronger signal from PIN4 in stele cells (Figure 8, Figure S3). 

Published data showed that prolonged salt stress (6 h) significantly reduced the transcript levels and fluorescence intensities of PIN1, PIN3, and PIN7 in roots, although their distribution patterns were not altered after 6 h of salt stress [34]. Previously, NaCl treatment was shown to strongly suppress the expression levels of PIN1, PIN2, PIN3, and DR5 after 5 days of treatment [41]. A decrease in auxin concentration in quiescent center cells may occur in other ways, by conjugation or degradation of auxin, or by efflux through other transporters. According to recent publications, certain genes involved in auxin homeostasis, such as IAR4, are induced to prevent NaCl overaccumulation [41]. The authors showed that NaCl accumulation promotes the production of ROS, which negatively modulates root growth by regulating PIN-mediated polar auxin transport in roots.

### 2.5. Effect of Stress Conditions on the Expression of Genes Involved in Auxin Metabolism 

**Figure 9:**
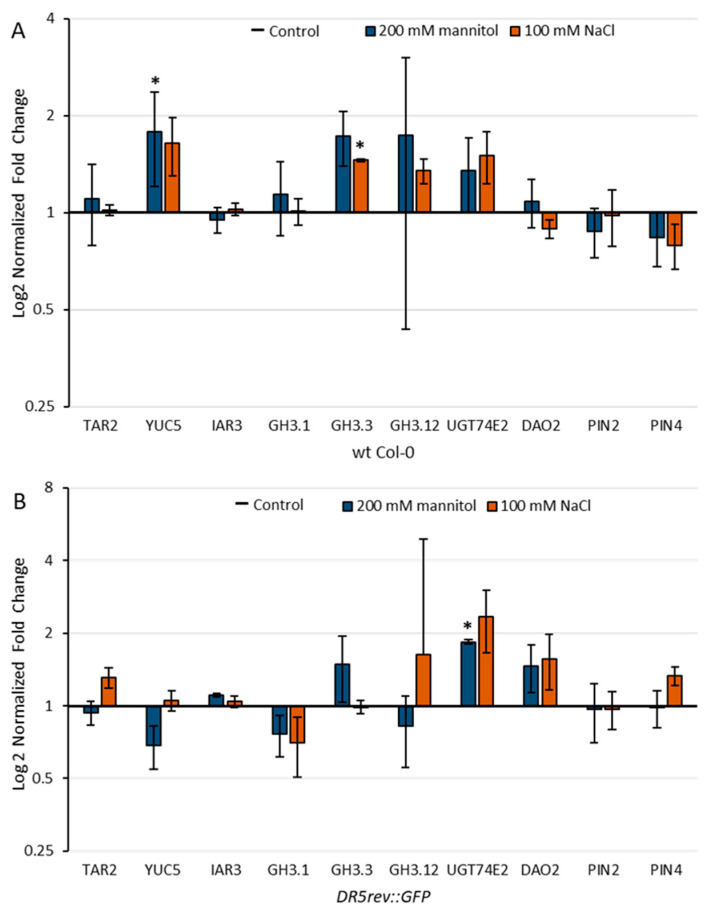
Relative gene expression of tryptophan aminotransferase related 2 (*TAR2*, At4g24670); flavin-binding monooxygenase family protein (*YUC5*, At5g43890); auxin amidohydrolase (*IAR3*, At1g51760); auxin amidosynthetase (*GH3.1*, At2g14960); auxin amidosynthetase (*GH3.3*, At2g23170); auxin amidosynthetase (*GH3.12*, At5g13320); uridine diphosphate glycosyltransferase 74E2 (*UGT74E2*, At1g05680); 2-oxoglutarate and Fe(II)-dependent oxygenase 2 (*DAO2*, At1g14120), and auxin transport proteins *PIN2* (At5g57090) and *PIN4* (At2g01420) in wild-type Arabidopsis Col-0 (**A**) and DR5 line (**B**) upon 3 h exposure to 100 mM NaCl or 200 mM mannitol compared to the control measured by qPCR. Gene expression was calculated according to Livak [45] and presented as fold change compared to the controls. Asterisk (*) indicates significant differences with respect to the corresponding control (Student’s *t* test, *p* < 0.05). Bars show means ± SE of three biological replicates.

IAA levels are tightly regulated by numerous processes such as IAA biosynthesis, ester- and amide-conjugation, conjugate hydrolysis, degradation, and auxin transport, as shown in Figure 1. For a deeper understanding of the observed changes in the auxin metabolome and auxin distribution under salinity and osmotic stresses, we analyzed the transcript levels of selected genes involved in auxin metabolism (tryptophan aminotransferase related 2 (*TAR2*, At4g24670); flavin-binding monooxygenase family protein (*YUC5*, At5g43890); auxin amidohydrolase (*IAR3*, At1g51760); auxin amidosynthetases (*GH3.1*, At2g14960; *GH3.3*, At2g23170; (*GH3.12*, At5g13320); uridine diphosphate glycosyltransferase 74E2 (*UGT74E2*, At1g05680); 2-oxoglutarate and Fe(II)-dependent oxygenase 2 (*DAO2*, At1g14120), and the auxin transport proteins *PIN2* (At5g57090) and *PIN4* (At2g01420) in the wild-type of the Col-0 ecotype and the DR5 line (Figure 9). The chromosomal locations for the selected genes studied are shown in Supplementary Materials Figure S5.

Targets for gene expression analysis were selected in accordance with the observed changes in our auxin profile and based on the transcriptome dataset [42] available on the eFP browser website [43]. Transcriptome dataset of the Arabidopsis Col-0 roots (stage 1.02, [44]) treated with 150 mM NaCl and 300 mM mannitol for the period of 0.5–24 h [42] was used to generate a heat map of auxin-related gene expressions (Supplementary Materials Table S1). The expression patterns of the above dataset showed that salinity stress induced more pronounced changes in auxin-related genes than osmotic stress. This is in general agreement with our auxin metabolome data (Figure 4). Certain differences in the expression of selected genes were observed among the Arabidopsis lines studied (Figure 9).

The transcript level of the gene *YUC5*, whose protein product is involved in the IPyA auxin biosynthetic pathway, was increased approximately 1.8- and 1.6-fold under osmotic and salinity stress, respectively, in the Col-0. However, in the DR5 line, expression was down-regulated under osmotic stress and slightly up-regulated under salinity stress compared with untreated controls. This observation is consistent with the auxin level measured in 4-day-old Arabidopsis seedlings in these two lines (Figure 4), which is generally lower in the DR5 line compared with Col-0. Based on the transcriptomic data, it appears that *YUC5* has a major role in Arabidopsis root auxin biosynthesis immediately after stressor application.

A similar effect with *YUC* genes has also been described in cucumber under salt stress, where *CsYUC10b* is strongly up-regulated and *CsYUC10a* and *CsYUC11* are strongly down-regulated. Moreover, overexpression of *CsYUC11* results in higher auxin levels in transgenic Arabidopsis and increases plant tolerance to salt stress as measured by root elongation [46]. Increased auxin levels in the plant have also been shown to increase the ability to cope with drought stress. Arabidopsis seedlings constitutively overexpressing *iaaM* and thus having higher endogenous auxin content showed higher resistance to drought stress compared to wild-type plants. Moreover, the *yuc1 yuc2 yuc6* triple mutant is more sensitive to drought than wild-type plants [47]. Similarly, transgenic poplar and potato plants overexpressing Arabidopsis *YUC6* showed increased resistance to abiotic stress [48,49]. The increased expression of the *YUC* gene, as well as the higher IAA level in Col-0 compared to DR5, correspond to more efficient lateral root induction of the Col-0 line than of the DR5 line under stress conditions (Figure 2 and Figure 3).

According to transcriptomic analysis, the most impaired process of auxin homeostasis under stress conditions was the synthesis of amide and ester conjugates (Supplementary Materials Table S1). It has been previously shown that GH3-mediated auxin homeostasis is an important part of the complex network of auxin actions that regulate stress adaptation in Arabidopsis [50]. Recently published work based on transcriptomic data and qRT-PCR results indicate that GH3s play important roles under salinity and osmotic stress in different plant species [51,52,53]. Importantly, the significant increase in IAA-Glu levels observed in Arabidopsis wild type roots stressed for 13-days (Figure 5) is consistent with the transcriptome data (Figure 9). Under our experimental conditions, *GH3.1*, *GH3.3,* and *GH3.12* were up-regulated by both stressors in Col-0, whereas DR5 surprisingly showed some differences in the expression of GH3s. In the stress-treated DR5 line, *GH3.3* was mainly up-regulated under osmotic stress, *GH3.12* under salinity stress, and *GH3.1* was down-regulated under both stressors compared with the untreated control seedlings. 

IAR3, the auxin amidohydrolase representative that can contribute to free IAA level by hydrolysis of amide conjugates, was slightly up-regulated in the stress treated DR5 line compared with the control. Kinoshita et al. [54] suggested that activation of IAR3 contributes to free auxin mediating root architecture under water limitation. It was also shown that the level of IAR3 transcript was increased in Arabidopsis due to high osmotic stress.

In addition, the *UGT74E2* gene involved in ester conjugation of IAA was overexpressed in both lines under stress conditions, but more clearly in the DR5 line. In a recent publication, UGT74E2 and UGT74D1, among others, were identified as UGT candidates that may increase tolerance to salinity and drought stress in Brassica and Arabidopsis [55]. However, we could not detect increased IAA-Glc level under stress conditions (Figure 4 and Figure 5) probably due to back-cleavage to the IAA.

DAO2 is involved with DAO1 in the degradation and oxidation of IAA to oxIAA. The *DAO2* gene is slightly up-regulated in the DR5 line under stress conditions compared with the control, although we did not detect increased levels of oxIAA in the seedlings under stress conditions. On the other hand, a reduction in oxIAA levels was also detected in Arabidopsis roots after 48 h exposure to salinity stress [37].

Most genes for transport proteins were slightly down-regulated under stress conditions (Supplementary Materials Table S1) [34,41]. Under our experimental conditions, the *PIN4* transcript level was increased in the DR5 line compared to the control, which is consistent with the stronger fluorescence signal observed under salt stress (Figure 8).

**Figure 10:**
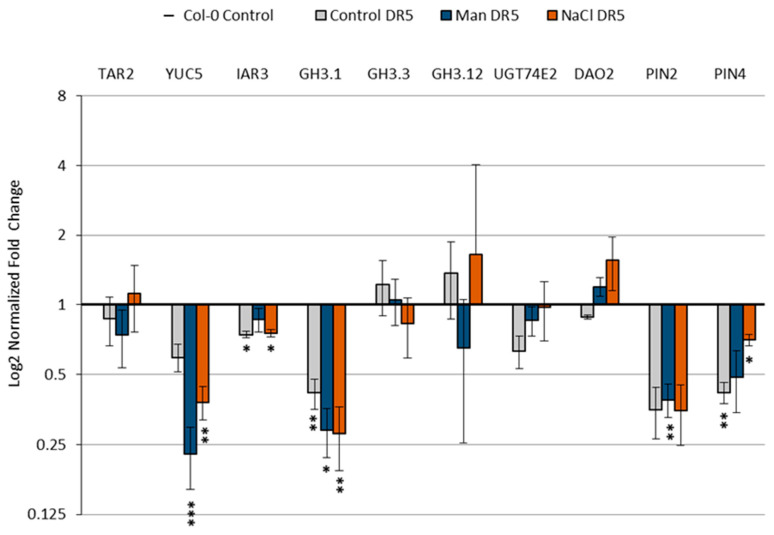
Relative gene expression in Arabidopsis DR5 (*DR5rev::GFP*) line upon 3 h exposure to 100 mM NaCl or 200 mM mannitol compared to the Col-0 in each of the tested conditions of: tryptophan aminotransferase related 2 (*TAR2*, At4g24670); flavin-binding monooxygenase family protein (*YUC5*, At5g43890); auxin amidohydrolase (*IAR3*, At1g51760); auxin amidosynthetase (*GH3.1*, At2g14960); auxin amidosynthetase (*GH3.3*, At2g23170); auxin amidosynthetase (*GH3.12*, At5g13320); uridine diphosphate glycosyltransferase 74E2 (*UGT74E2*, At1g05680); 2-oxoglutarate and Fe(II)-dependent oxygenase 2 (*DAO2*, At1g14120), and auxin transport proteins *PIN2* (At5g57090) and *PIN4* (At2g01420). Gene expression was calculated according to Livak [45] and presented as fold change compared to Col-0. Asterisk (*) indicates significant differences with respect to the Col-0 growth conditions as the control (Student’s *t* test, * presents *p* < 0.05, ** *p*< 0.01, *** *p* < 0.001). Bars show means ± SE of three biological replicates.

Figure 10 shows the comparison of selected gene expressions between Col-0 and DR5. 

It can be seen that the transcript levels of most of the genes examined were lower in the DR5 line compared to Col-0 (*TAR2*, *YUC5*, *IAR3*, *GH3.1*, *UGT74E2*, *PIN2,* and *PIN4*). Only *DAO2* showed higher expression in the DR5 line under stress conditions. Interestingly, we observed that *GH3.3* and *GH3.12* showed higher transcript levels in the DR5 line compared with the Col-0 under control conditions, which is in agreement with higher levels of amide conjugates (IAA-Asp and IAA-Glu) in DR5 control seedlings compared with Col-0 control seedlings. In conclusion, higher transcript levels of *GH3s* and *DAO2* genes in the DR5 line indicate that this line is more sensitive than Col-0 under stress conditions, consistent with the root growth assay (Figure 2 and Figure 3).

The qPCR data are consistent with the auxin profile and root growth assay results in both lines (Figure 2, Figure 3 and Figure 5). Although the DR5 line appeared to be more sensitive compared with the wild-type Col-0 under our experimental conditions, similar trends of the morphological, metabolome and transcriptome parameters were observed in both lines under both stress conditions. Both stressors caused significant inhibition in main root growth as well as LRs number and total LRs length in both lines compared to their corresponding controls. Moreover, a similar trend of IAA increase was observed in both lines under both stress conditions and amide conjugation was strongly induced under both stress conditions. By taking all these into consideration, we would conclude that the DR5 line shows some aberrations in lateral root production under salt and osmotic stress but it is a useful and reliable tool for visualizing auxin distribution under different experimental setups.

## 3. Materials and Methods

### 3.1. Reagents and Standards

Murashige and Skoog (MS) powder medium containing vitamins, plant agar, and 2-(N-morpholino)ethanesulfonic acid (MES) monohydrate were obtained from Duchefa, Haarlem, The Netherlands. Sucrose, NaCl, mannitol were purchased from Kemika, Zagreb, Croatia, while Izosan G was purchased from Pliva, Zagreb, Croatia. Propidium iodide (PI) was obtained from Sigma-Aldrich, St. Louis, MO, USA. Standards for IAA and its precursors anthranilate (ANT), indole-3-acetaldehyde (IAAld), IAM, indole-3-acetonitrile (IAN), IPyA, tryptamine (TRA), and tryptophan (TRP) were purchased from Sigma-Aldrich, and standards for IAOx and 2-oxoindole-3-acetic acid (oxIAA) from OlChemIm Ltd, Olomouc, Czech Republic. Acetic acid was purchased from Merck (Darmstadt, Germany), diethyldithiocarbamic acid sodium salt and cysteamine hydrochloride from Sigma-Aldrich, St. Louis, MO, USA, and HPLC gradient grade solvents from J.T. Baker—Fisher Scientific, Hampton, NH, USA. All other chemicals were from Carl Roth, Karlsruhe, Germany and Sigma-Aldrich.

### 3.2. Plant Material and Growth Conditions

Seeds of Arabidopsis (*Arabidopsis thaliana*) ecotype Columbia-0 and the previously described *DR5rev::GFP* line (N9361) [56,57] were purchased from The Nottingham Arabidopsis Stock Centre [58]. Seeds of the transgenic Arabidopsis lines *pPIN2::PIN2-GFP* [59], *pPIN4::PIN4-GFP* [60], *pPIN7::PIN7-GFP* [61] were kindly provided by C. Luschnik and J. Friml.

Seeds were surface sterilized with 70% ethanol (*v*/*v*) for 1 min, followed by 1% Izosan G (*w*/*v*) for 10 min, washed five times with sterile distilled water, seeded on the full-strength MS medium containing vitamins, 1% (*w*/*v*) plant agar, 1% (*w*/*v*) sucrose, 2,5 mM MES monohydrate, (pH 5.7). After stratification for 2 days at 4 °C, plates were transferred to a growth chamber (PHC Corporation, Tokio, Japan), positioned vertically, at 22 °C under 16 h of light at 150 μmol m^−2^ s^−1^/8 h darkness. Four-day-old seedlings were used for further experiments.

### 3.3. Stress Treatments of Seedlings

To investigate the effect of the stressors (sodium chloride and mannitol) long-term (up to 13 days) short-term (1.5 to 12 h) and immediate (0 to 15 min) analyses of auxin synthesis, accumulation and distribution were performed. For long-term stress application 4-day-old seedlings germinated on MS agar plates supplemented with 2.5 mM MES buffer (pH 5.7) were transferred to the fresh plates of the same medium (Control) or to the same medium supplemented with either 100 mM NaCl (NaCl) or 200 mM mannitol (Man). Seedlings were further cultured for 13 days at 22 °C under 16 h of light at 150 μmol m^−2^ s^−1^/8 h darkness. For short-term stress application 4-day-old seedlings germinated on MS agar plates supplemented with 2.5 mM MES buffer (pH 5.7) were transferred to 2.5 mM MES buffer (pH 5.7) (Control) or the same buffer supplemented with either 100 mM NaCl (NaCl) or 200 mM mannitol (Man). For immediate stress application, 4-day-old seedlings germinated on MS agar plates containing 2.5 mM MES buffer (pH 5.7) were transferred to a microscopic slide with a drop of distilled water and observed. Distilled water was replaced by adding an excessive volume of 100 mM NaCl soaked with filter paper under the coverslip and the seedlings were immediately further analyzed. 

For gene expression analysis, the roots of 4-day-old seedlings were directly covered on Petri dishes with 15 mL of either 2.5 mM MES buffer (pH 5.7) as a control or with 100 mM NaCl in 2.5 mM MES buffer or 200 mM mannitol in 2.5 mM MES buffer and placed horizontally in the growth chamber for 3 h. After the treatments, roots were excised, frozen with liquid nitrogen, and stored at −80 °C until RNA isolation.

### 3.4. Root Growth Bioassay 

To investigate the effect of stressors on the root growth rate and the root architecture long-term stress was applied. Root growth bioassay was performed on Arabidopsis ecotype Columbia-0 (Col-0) and the *DR5rev::GFP* (DR5) lines. Four-day-old seedlings were transferred to media containing stressors or control medium and were grown for the next 13 days on vertically positioned plates in the growth chamber under previously described conditions, and periodically observed for root growth and architecture. Plates were then scanned byHP Scanjet G3010 scanner (Hewlett-Packard, Palo Alto, CA, USA) and root images analyzed using the EZ Rhizo II program (v2.5.0.4, University of Glasgow, Glasgow, UK) [62].

### 3.5. Confocal Laser Scanning Microscopy and Image Analyses

Auxin induced GFP synthesis was followed by confocal microscopy in root cells of 4-day-old DR5 seedlings that were exposed to short-term stress treatment. Seedlings were exposed to the stressors for 3 and 6 h. Based on the preliminary results showing a drastic redistribution of auxin upon short-term salinity stress (3 h), we further investigated localizations of PINs (PIN2, PIN4, and PIN7) upon immediate salinity stress (5, 10, or 15 min). 

After short-stress treatments, DR5 seedlings were stained in 50 µgmL^−1^ propidium iodide (PI) solution for 1 min, rinsed in distilled water, and mounted in water on microscope slides. Images were acquired in 8-bit format using a Leica TCS SP8 X laser scanning confocal microscope with 40× numerical aperture (NA) = 1.3 oil-immersion objective and a pinhole corresponding to 1.0× the diameter of Airy disk or with Leica TCS SP2 laser scanning confocal microscope with 20× numerical aperture (NA) = 0.70 and 63× NA = 1.40 oil-immersion objective. The system was controlled with the Leica Application Suite X Software (v3.1.1.15751, Leica Microsystems, Wetzlar, Germany) or with Leica Confocal Software LCS (v2.6.0, Leica Microsystems, Wetzlar, Germany). GFP and PI were excited at 488 nm by a visible gas Argon laser. GFP emission was detected at 500–530 nm (or 500–563 nm), while PI was detected at 597–670 nm. All confocal images were acquired with a constant set of microscopy, detection, and excitation parameters. At least ten independent seedlings were analyzed per treatment, and treatment representative images were selected for figure construction. For the acquisition of a Z-stack image sequence, images were acquired in 20 focal planes with 0.5 μm z-spacing, 30 nm xy-pixel size, and 400 Hz unidirectional xyz scan mode. For the measurement of fluorescence intensity, optical sections at the focal plane of the quiescent center cells were acquired. Fluorescence intensity was quantified according to Gu et al. [63] using the program Fiji-ImageJ (v1.51n Java 1.8.0_66, National Institutes of Health, Bethesda, MD, USA) [64]. Ten roots per treatment were examined, and at least three independent experiments were performed. 

### 3.6. Auxin Metabolite Analysis

Auxin metabolome was assessed in young 4-day-old seedlings of Col-0 and the DR5 line treated with either 100 mM NaCl or 200 mM mannitol in 2.5 mM MES buffer (pH 5.7) for 3 h. Untreated seedlings grown on 2.5 mM MES buffer (pH 5.7) were used as controls. Furthermore, the roots of Arabidopsis plants were also analyzed after prolonged stress conditions (13-day treatments). After the long-term treatment, four to five biological replicates of plant samples were collected and weighed up to 10 mg fresh weight per replicate. Complete auxin profiling was performed using the ultra-rapid auxin metabolite profiling method, previously described [65]. All purified samples were analyzed by a 1260 Infinity LC system combined with a 6495 Triple Quadrupole LC/MS system (Agilent Technologies, Santa Clara, CA, USA) using stable isotope-labeled internal standards as a reference. 

### 3.7. Real-Time PCR of Selected Genes

Each sample (biological replicate) for RNA isolation contained control or stress treated roots from 75 seedlings that were frozen in liquid nitrogen and stored at −80 °C. For total RNA isolation, 500 µL of RNAzol (Sigma Aldrich, St. Louis, MO, USA) was added to the sample, after which the roots were homogenized on the Mixer Mill MM400 (Retsch GmbH, Haan, Germany) in three cycles of 1 min at 30 Hz with zirconium oxide beads (Next Advance, Troy, NY, USA). After centrifugation, an equal volume of ethanol (100%) was added to the supernatants, which were further processed using the Direct-zol^TM^ RNA MiniPrep kit (Zymo Research, Irvine, CA, USA) according to the manufacturer’s instructions. DNA was removed with recombinant RNase-free Dnase I (Roche Diagnostics GmbH, MannheimGermany). Up to 30 μL of total RNA (1 µg) was incubated with 1U of DNase for 30 min at 37 °C in 1X incubation buffer, followed by the addition of 250 µL of Binding Buffer from GeneJET RNA Cleanup and Concentration Micro Kit (ThermoScientific, Waltham, MA, USA) and subsequent purification according to the manufacturer’s instruction. The final concentrated DNase treated RNA was eluted in 10 µL of RNase free water. RNA integrity was checked before and after DNase I treatment by measuring the absorbance with NanoDrop ND-2000 spectrophotometer (NanoDrop Technologies, Wilmington, DE, USA) and by non-denaturing agarose gel-electrophoresis. cDNA was synthesized using Maxima First Strand cDNA Synthesis Kit for qPCR (ThermoScientific, Waltham, MA, USA) according to the manufacturer’s protocol. Reverse transcription was performed with 0.3 μg of DNase treated total RNA in a thermocycler (Applied Biosystems, Waltham, MA, USA) under the following conditions: 10 min at 25 °C, followed by 15 min at 50 °C and 5 min at 85 °C.

Gene-specific primers were designed using Quant Prime [66] and Primer-BLAST [67] and the reference gene was selected according to Czechowski et al. [68]. All the primers used are listed in Supplemental Material Table S2. Quantitative PCR was performed in a 96-well optical plate using the 7300 Real-Time PCR system (Applied Biosystems) and universal cycling conditions (10 min 95 °C, 40 cycles of 15 s at 95 °C and 60 s at 60 °C) followed by the generation of a dissociation curve to check for the specificity of the amplification. Reactions contained Power SYBR Green Master Mix (Applied Biosystems), 200 nM of a gene-specific forward and reverse primer, and 3 μL of 10x diluted cDNA in each 15 μL reaction. No template controls (NTC) contained 3 μL RNase free water instead. For qPCR, three biological replicates were performed in triplicate (technical replicates). PCR amplification efficiencies were calculated using LinRegPCR software v2020.2 [69]. Supplemental Material Table S2 shows the amplification efficiencies for the primer pairs we studied. Relative expression (fold change) was calculated using the comparative 2^−ΔΔCt^ method [45] with the TIP41- like gene (AT4G34270) as the reference gene. Statistical analysis of the obtained data was performed using Student’s *t*-test to detect the differences between control and treated groups. The results are presented as mean ± SE of three independent experiments. The standard error was calculated using the results of the 2^−ΔΔCt^ method. The chromosomal locations for the studied genes have been depicted based on data from TAIR10 assembly by Chromosome viewer at bar.utoronto.ca/eplant [70].

### 3.8. Statistical Analysis

Significance of differences was determined using Student’s test, as indicated in figures (*, **, and *** correspond to *p*-values of 0.05 > *p* > 0.01, 0.01 > *p* > 0.001, and *p* < 0.001, respectively). Each data point indicated represents the average of three replicates (*n* = 3) unless otherwise stated.

## 4. Conclusions

We examined the effect of short-term (3 h) and prolonged (13 days) salt (100 mM NaCl) and osmotic (200 mM mannitol) stress on the auxin metabolome, auxin distribution, and transcript levels of selected auxin related genes in Arabidopsis Col-0 and DR5 line. Both abiotic stresses caused inhibition of main root growth and lateral root development in response to treatment duration, with DR5 lines being more affected compared to Col-0. Prolonged osmotic stress caused more drastic changes in root growth and LRs development compared with salt stress, probably due to the ability of plants to neutralize NaCl by compartmentalization. Short-term (3 h) stress caused significant changes in the auxin metabolome, especially the NaCl treatment, which resulted in a significant IAA decrease compared with the control. This is in agreement with the auxin distribution observed by confocal microscopy in the DR5 line. NaCl treatment resulted in a redistribution of auxin signaling from the quiescent center and the internal layers of the root cap (common to control and mannitol-treated roots) to the epidermal and cortical cells of the root elongation zone. PIN protein distribution and gene expression were also affected by stress conditions. PINs, especially PIN2 protein concentration were reduced during the first 5 min of salt treatment. This observation was also consistent with the auxin related transcriptome data from Col-0, suggesting disruption of auxin biosynthesis genes expression, but particularly amide and ester conjugation processes. Indeed, analysis of the transcription level of selected genes involved in auxin metabolism mainly confirmed higher transcription of *YUC*, *GH3s*, and *UDP* genes in both lines, whereas transcription of *PINs* was lower under stress conditions. Certain differences were observed between the Col-0 and DR5 lines, which showed a lower transcription level of *YUC5* and *PINs*. In contrast, the transcription level of *GH3* genes and *DAO2* was higher in the DR5 line than in the Col-0. Moreover, our gene expression data correspond to the differences in the auxin metabolome determined between the two lines. Interestingly, Col-0 seedlings contained more IAA, whereas DR5 seedlings had higher levels of the amide conjugates IAA-Glu and IAA-Asp. During prolonged stress conditions, the auxin metabolome showed an intention to stabilize, and a tendency to increase IAA was observed, especially under salt stress. 

In conclusion, salinity stress, a complex stress composed of the toxic and osmotic effects of salt, causes more striking changes in the distribution of auxin as well as in its metabolome and transcriptome during a short treatment. However, a longer stress treatment with NaCl caused less conspicuous changes in root growth and architecture, while IAA content increased more frequently. Based on the results under our experimental conditions, the DR5 line, which is commonly used for auxin monitoring, appeared to be more sensitive to the applied stresses than the wild type Col-0, although the trends in morphological, metabolome, and transcriptome parameters are similar. To our opinion, this observation is useful and new information that may help researchers who use the DR5 auxin reporter line in explanations of maybe unexpected results. As far as adequate control is included there is no doubt that this line is a very useful and helpful research tool in the visualization of auxin distribution.

## Supplementary Materials

The following are available online at https://www.mdpi.com/article/10.3390/ijms22157993/s1, Figure S1: Seedlings morphology of *Arabidopsis thaliana* Columbia-0 (Col 0-WT) and *DR5rev::GFP* line (DR5) grown on MS medium (control) or treated with 100 mM NaCl (NaCl) or 200 mM mannitol (Man) for 13 days. Figure S2: PIN2 proteins distribution in the root tips of Arabidopsis *pPIN2::PIN2-GFP* line in response to salt stress (100 mM NaCl) and corresponding controls in time range 5–15 min. Figure S3: PIN4 proteins distribution in the root tips of Arabidopsis *pPIN4::PIN42-GFP* line in response to salt stress (100 mM NaCl) and corresponding controls in time range 5–15 min. Figure S4: PIN7 proteins distribution in the root tips of Arabidopsis *pPIN74::PIN47-GFP* line in response to salt stress (100 mM NaCl) and corresponding controls in time range 5–15 min. Figure S5. The chromosomal locations for the genes studied in Figure 9 and Figure 10 based on the genome assembly of *Arabidopsis thaliana* visualized by Chromosome viewer at bar.utoronto.ca/eplant [70]. Table S1: Micro-array data in salt stress (150 mM NaCl) and osmotic stress (300 mM mannitol) in time range 0.5–24 has been published according to Killian et al. [42] for genes involved in auxin metabolism. The data has been derived from the eFP browser website [43], and the normalization has been used as published on this website. IAA metabolism is illustrated in Figure 1. Relative values represent log2 fold change of expression upon salt and mannitol treatments. The expression level is marked by gradation of color intensity: from bright to dark blue (decrease in expression), over white (unaltered expression), from bright to dark red (increase in expression). Table S2: qPCR primer list and corresponding amplification efficiencies as calculated by Ruijter et al. [69]. Video S1: Z-stack of *DR5rev::GFP* mock treated root made by laser scanning confocal microscopy as described in Materials and Methods. A stack of 20 images (0.5 µm spacing) of optical sections around the quiescent center of roots mock treated for 3 h (in 1xMS, 2.5 mM MES, pH 5.7 supplemented with water as mock treatment). Z-stack was depicted in ImageJ as a video of a combined stack of three channels showed from left to right as GFP channel (GFP signal transformed with LUT Fire option), bright field channel (middle), and merged channel (furthest right). Scale bar- 50 µm, calibration bar—range of pixel intensities from 0–255. Video S2: Z-stack of *DR5rev::GFP* mannitol treated root made by laser scanning confocal microscopy as described in Materials and Methods. A stack of 20 images (0.5 µm spacing) of optical sections around the quiescent center of roots treated with mannitol for 3 h (in 1xMS, 2.5 mM MES, pH 5.7 supplemented with 200 mM mannitol). Z-stack was depicted in ImageJ as a video of a combined stack of three channels showed from left to right as GFP channel (GFP signal transformed with LUT Fire option), brightfield channel (middle), and merged channel (furthest right). Scale bar- 50 µm, calibration bar—range of pixel intensities from 0–255. Video S3: Z-stack of *DR5rev::GFP* salt treated root made by laser scanning confocal microscopy as described in Materials and Methods. A stack of 20 images (0.5 µm spacing) of optical sections around the quiescent center of roots treated with NaCl for 3 h (in 1xMS, 2.5 mM MES, pH 5.7 supplemented with either 100 mM NaCl). Z-stack was depicted in ImageJ as a video of a combined stack of three channels showed from left to right as GFP channel (GFP signal transformed with LUT Fire option), bright field channel (middle), and merged channel (furthest right). Scale bar- 50 µm, calibration bar—range of pixel intensities from 0–255.
