# Supplementary material for: Altered Root Growth, Auxin Metabolism and Distribution in Arabidopsis thaliana Exposed to Salt and Osmotic Stress
Source: Int J Mol Sci. 2021 Jul 27;22(15):7993. doi: 10.3390/ijms22157993 (PMC8348202; doi:10.3390/ijms22157993)
Supplement: Supplementary file 1 [file ijms-22-07993-s001.zip › Supplemental files/Supplemental Figures.pdf]

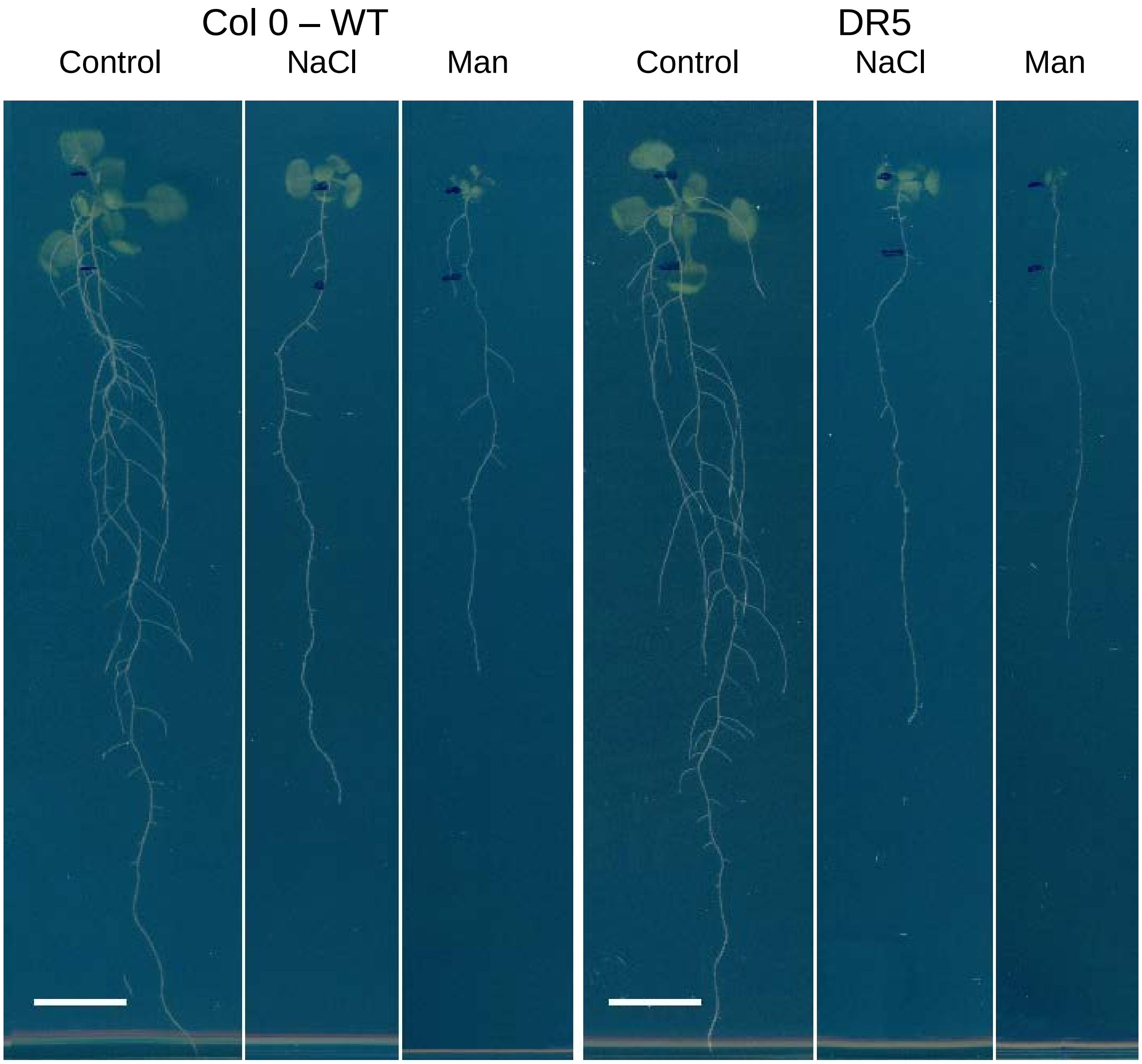

Figure S1. Seedlings morphology of *Arabidopsis thaliana* Columbia-0 (Col 0-WT) and *DR5rev::GFP* line (DR5) grown on MS medium (control) or treated with 100 mM NaCl (NaCl) or 200 mM mannitol (Man) for 13 days. Bar: 10 mm.

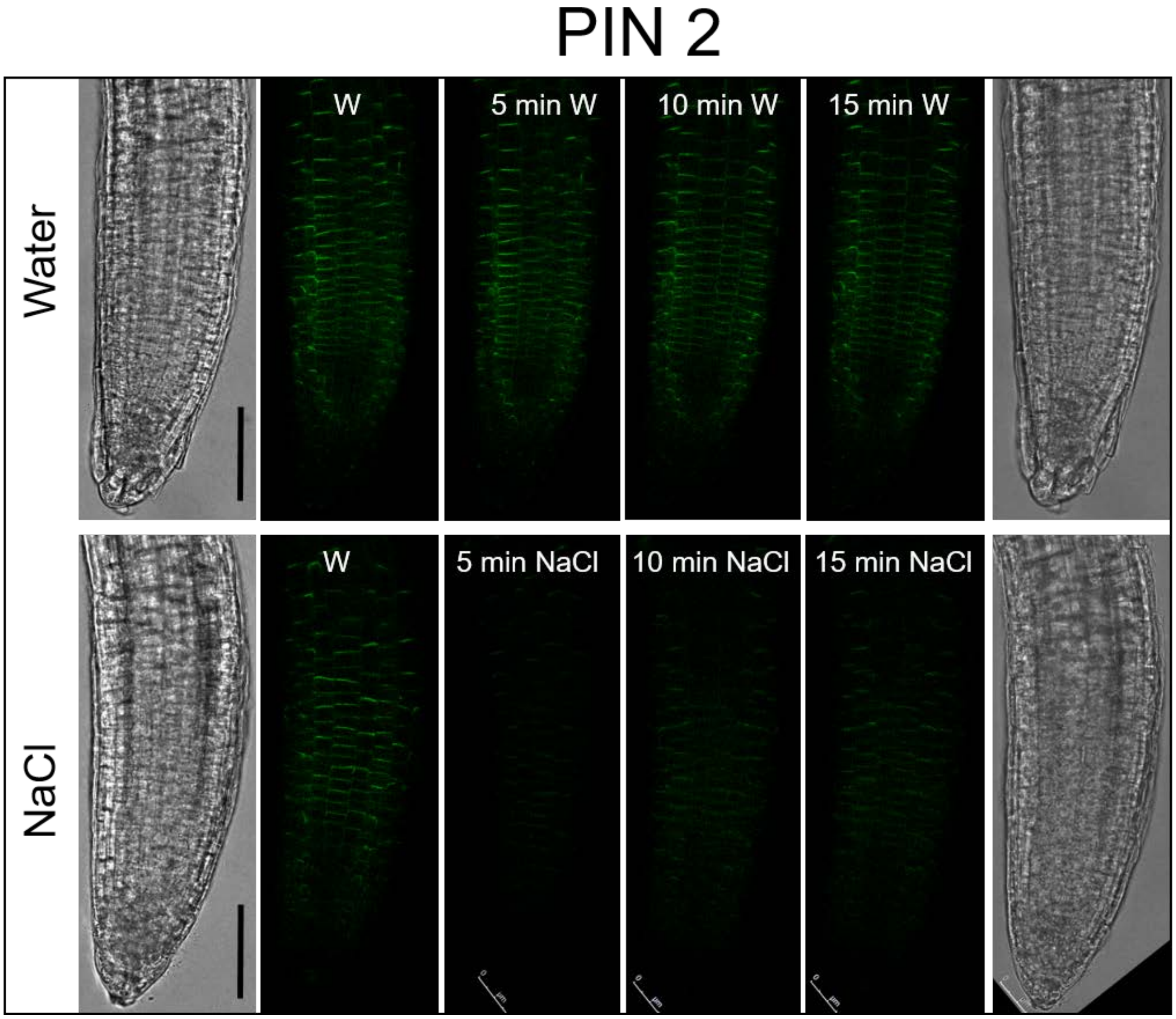

Figure S2. PIN2 proteins distribution in the root tips of Arabidopsis *pPIN2::PIN2-GFP* line in response to salt stress (100 mM NaCl) and corresponding controls in time range 5-15 min. GFP fluorescence was imaged with a Leica TCS SP8 X laser scanning confocal microscope. Bar: 50 µm.

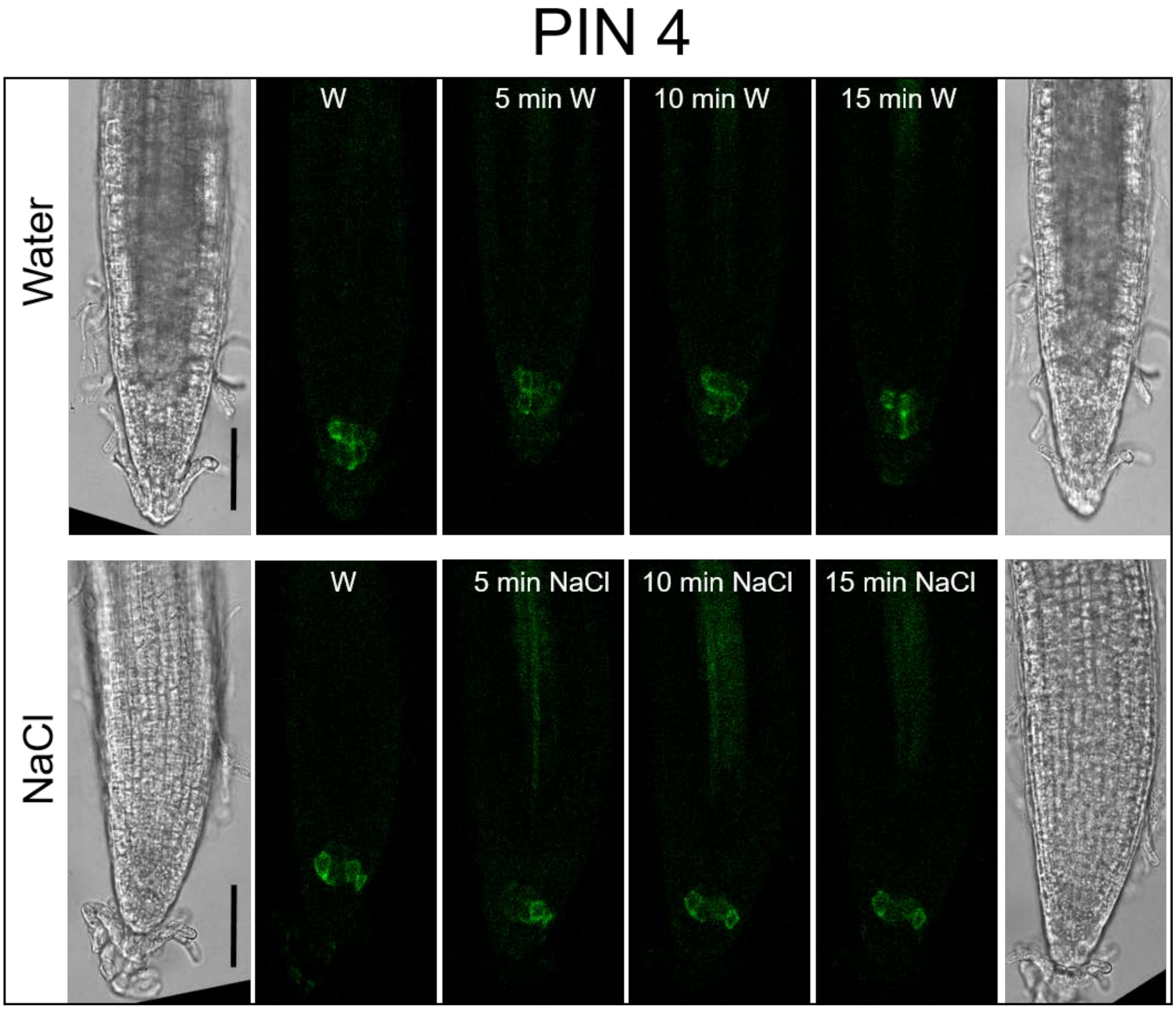

Figure S3. PIN4 proteins distribution in the root tips of Arabidopsis *pPIN4::PIN42-GFP* line in response to salt stress (100 mM NaCl) and corresponding controls in time range 5-15 min. GFP fluorescence was imaged with a Leica TCS SP8 X laser scanning confocal microscope. Bar: 50 µm.

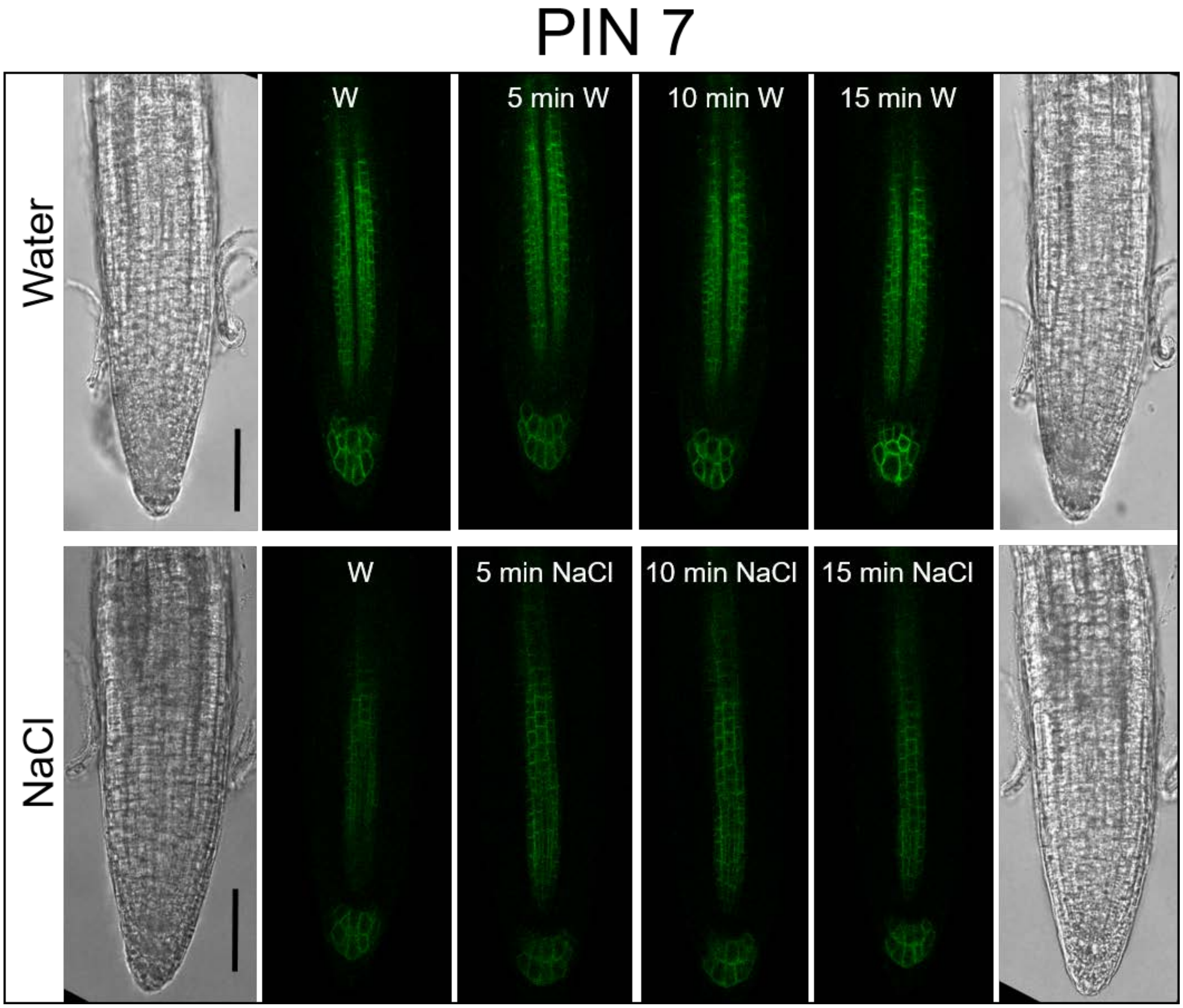

Figure S4. PIN7 proteins distribution in the root tips of Arabidopsis *pPIN74::PIN47-GFP* line in response to salt stress (100 mM NaCl) and corresponding controls in time range 5-15 min. GFP fluorescence was imaged with a Leica TCS SP8 X laser scanning confocal microscope. Bar: 50 µm.

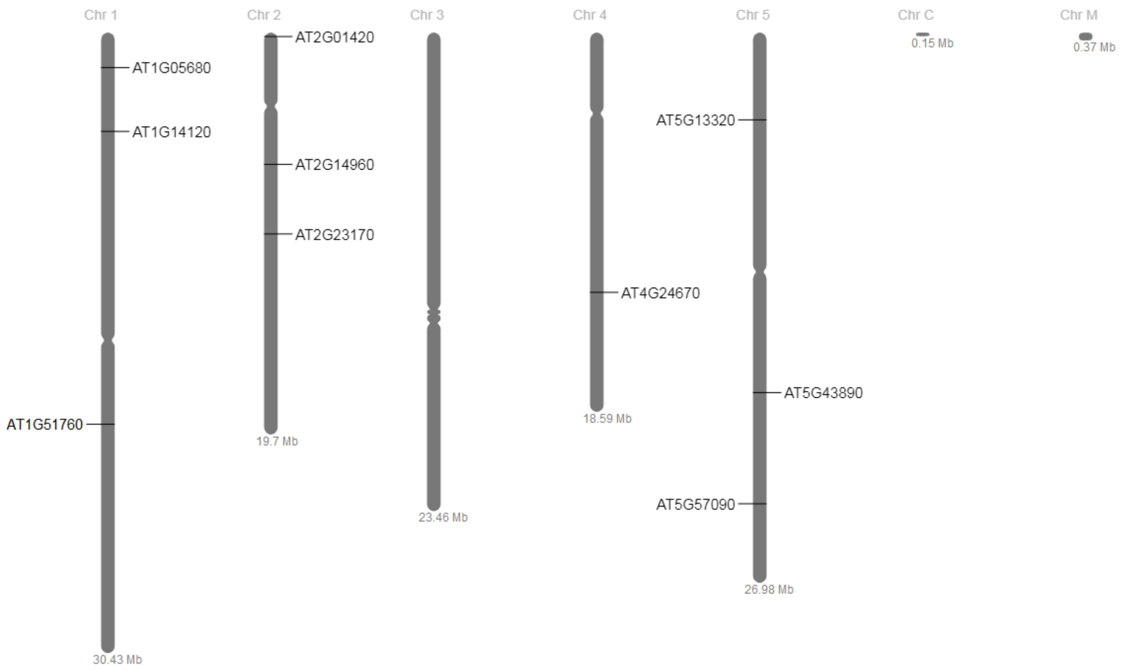

Figure S5. The chromosomal locations for the genes studied in Fig. 9 and Fig. 10 based on the genome assembly of *Arabidopsis thaliana*: tryptophan aminotransferase related 2 (TAR2, At4g24670); flavin-binding monooxygenase family protein (YUC5, At5g43890); auxin amidohydrolase (IAR3, At1g51760); auxin amidosynthetase (GH3.1, At2g14960); auxin amidosynthetase (GH3.3, At2g23170); auxin amidosynthetase (GH3.12, At5g13320); uridine diphosphate glycosyltransferase 74E2 (UGT74E2, At1g05680); 2-oxoglutarate and Fe(II)-dependent oxygenase 2 (DAO2, At1g14120), and auxin transport proteins PIN2 (At5g57090) and PIN4 (At2g01420). Data for this view come from TAIR10 assembly. This image was generated with the Chromosome viewer at bar.utoronto.ca/eplant by Waese et al. [70].
