# Supplementary material for: Altered Root Growth, Auxin Metabolism and Distribution in Arabidopsis thaliana Exposed to Salt and Osmotic Stress
Source: Int J Mol Sci. 2021 Jul 27;22(15):7993. doi: 10.3390/ijms22157993 (PMC8348202; doi:10.3390/ijms22157993)
Supplement: Supplementary file 1 [file ijms-22-07993-s001.zip › Supplemental files/Supplemental Videos/Supplemental videos-legend.pdf]

Video S1. Z-stack of *DR5rev::GFP* mock treated root made by laser scanning confocal microscopy as described in Materials and Methods. A stack of 20 images (0.5 µm spacing) of optical sections around quiescent centre of roots mock treated for 3 hours (in 1xMS, 2.5 mM MES, pH 5.7 supplemented with water as mock treatment). Z-stack was depicted in ImageJ as a video of a combined stack of three channels showed from left to right as GFP channel (GFP signal transformed with LUT Fire option), bright field channel (middle), and merged channel (furthest right). Scale bar- 50 µm, calibration bar - range of pixel intensities from 0- 255.

Video S2. Z-stack of *DR5rev::GFP* mannitol treated root made by laser scanning confocal microscopy as described in Materials and Methods. A stack of 20 images (0.5 µm spacing) of optical sections around quiescent centre of roots treated with mannitol for 3 hours (in 1xMS, 2.5 mM MES, pH 5.7 supplemented with 200 mM mannitol). Z-stack was depicted in ImageJ as a video of a combined stack of three channels showed from left to right as GFP channel (GFP signal transformed with LUT Fire option), bright field channel (middle), and merged channel (furthest right). Scale bar- 50 µm, calibration bar - range of pixel intensities from 0- 255.

Video S3. Z-stack of *DR5rev::GFP* salt treated root made by laser scanning confocal microscopy as described in Materials and Methods. A stack of 20 images (0.5 µm spacing) of optical sections around quiescent centre of roots treated with NaCl for 3 hours (in 1xMS, 2.5 mM MES, pH 5.7 supplemented with either 100 mM NaCl). Z-stack was depicted in ImageJ as a video of a combined stack of three channels showed from left to right as GFP channel (GFP signal transformed with LUT Fire option), bright field channel (middle), and merged channel (furthest right). Scale bar- 50 µm, calibration bar - range of pixel intensities from 0- 255.
